# Supplementary material for: Host–Pathogen Interactions in Leaf Petioles of Common Ash and Manchurian Ash Infected with Hymenoscyphus fraxineus
Source: Microorganisms. 2022 Feb 5;10(2):375. doi: 10.3390/microorganisms10020375 (PMC8875166; doi:10.3390/microorganisms10020375)
Supplement: Supplementary file 1 [file microorganisms-10-00375-s001.zip › microorganisms-1544637-supplementary.pdf]

Supplemental Table S1. Identification and field performance of common ash clones and Manchurian ash clones (native host) used in the study. PDS: Percent damage score of the crown. During field assessments, crown damage is scored in classes and class mean averages across five years are given in the table. For Tapsøre the score is an average across the years 2011-2015 and for Randers from 2013-2017. The Manchurian ash from the Arboretum Hørsholm was only scored in 2013.

Clone Name	Trial	ID	Coordinate position in field trials	PDS (%)	Classification in field
35	FP281 Tapsøre	35	08;03	5	Less susceptible
14165	F384 Randers	S14165	14;08	8	Less susceptible
40	FP281 Tapsøre	40	08;17	34	Susceptible
27x	FP281 Tapsøre	27x	01;17	18	Susceptible
1989-0095-4	Arboretum Hørsholm	1989-0095-4	1603;1511	0	Less susceptible
